# CLEC10A is a prognostic biomarker and correlated with clinical pathologic features and immune infiltrates in lung adenocarcinoma

**DOI:** 10.1111/jcmm.16416

**Published:** 2021-03-02

**Authors:** Min He, Ying Han, Changjing Cai, Ping Liu, Yihong Chen, Hong Shen, Xingyuan Xu, Shan Zeng

**Affiliations:** ^1^ Department of Oncology Xiangya Hospital Central South University Changsha China; ^2^ Key Laboratory for Molecular Radiation Oncology of Hunan Province Xiangya Hospital Central South University Changsha China; ^3^ National Clinical Research Center for Geriatric Disorders Xiangya Hospital Central South University Changsha China; ^4^ Department of General Surgery Liaoning Cancer Hospital & Institute Shenyang China

**Keywords:** bioinformatics, biomarkers, CLEC10A, immuneinfiltrate, LUAD

## Abstract

CLEC10A, (C‐type lectin domain family 10, member A), as the member of C‐type lectin receptors (CLRs), plays a vital role in modulating innate immunity and adaptive immunity and has shown great potential as an immunotherapy target for cancers. However, there is no functional research of CLEC10A in prognostic risk, immunotherapy or any other treatment of lung adenocarcinoma (LUAD). We performed bioinformatics analysis on LUAD data downloaded from TCGA (The Cancer Genome Atlas) and GEO (Gene Expression Omnibus), and jointly analysed with online databases such as HPA, LinkedOmics, TIMER, ESTIMATE and TISIDB. We found that lower expression of CLEC10A was accompanied with worse outcomes of LUAD patients. Moreover, CLEC10A expression was significantly correlated with a variety of the tumour‐infiltrating immune cells (TIICs). As a promising prognosis predictor and potential immunotherapy target, the potential influence and mechanisms of CLEC10A in LUAD deserve further exploring.

**Abbreviations**
CLEC10A: C‐type lectin domain family 10, member A
CLRs: C‐type lectin receptors
ECM: extracellular matrix
GEO: Gene Expression Omnibus
GO: Gene Ontology
KEGG: Kyoto Encyclopedia of Genes and Genomes
LUAD: lung adenocarcinoma
MGL: macrophage galactose type C‐type lectin
MMP: matrix metalloproteinases
TAMs: tumour‐associated macrophages
TCGA: the Cancer Genome Atlas
TIICs: tumour‐infiltrating immune cells
TME: tumour microenvironment

## 1 INTRODUCTION

The morbidity and mortality of lung cancer were both increasing,^1^ among which lung adenocarcinoma (LUAD) was a common histological subtype.^2^ Despite the progresses made in cancer‐related treatment technology during the past years, the five‐year survival rate for lung cancer was still extremely poor, mainly because most patients are diagnosed at a last stage.^3^ Generally, the five‐year overall survival rate for patients diagnosed at advanced LUAD was just 15%; however, more than 60% of LUAD patients missed targetable gene alterations period, which could improve their survival rate.^4^, ^5^ Thus, discovering specific early detection markers and therapeutic targets is the key to improve survival rate of LUAD patients.

The level of immune cell infiltration in the tumour microenvironment (TME) plays a cardinal role in the tumour initiation, progression, metastasis and treatment resistance of cancer.^6^, ^7^, ^8^

CLEC10A, (C‐type lectin domain family 10, member A), is also named MGL, (macrophage galactose type C‐type lectin). As a member of the CLRs, CLEC10A, like other members, has been determined to involve in improving the immune response activity of immune cells. CLEC10A recognizes and acts on tumour‐associated Tn antigens and effectively presents the antigens to CD4 T cells.^9^ Furthermore, the binding of CLEC10A to tumour‐associated antigens carrying α‐N‐acetylgalactosamine can obviously increase antigen‐specific CD8 T cell activation.^10^ Effective tumour eradication requires tumour‐specific CD8 and CD4 T cells. The CLEC10A’ s function in improving the anti‐tumour activity of immune cells has clearly received people's attention and proposed it as a target for cancer immunotherapy.^11^

In this study, we conducted a comprehensive analysis of CLEC10A expression in the risk of LUAD progress based on more than 1200 patients, and then correlated CLEC10A different expression level and the alteration of tumour immune microenvironment. The results revealed the significant prognostic value of CLEC10A expression and a potentially promising target for immunotherapeutic strategies in LUAD.

## 2 MATERIALS AND METHODS

### 2.1 Data acquisition and processing

We downloaded the RNA expression profiles (RNA‐Seq2 level 3 data; platform: Illumina HiSeq 2000, through December 2019) and clinical data of LUAD patients from TCGA database (https://portal.gdc.cancer.gov/). TCGA provided 437 LUAD samples containing prognostic information and 54 normal lung tissue samples.

The gene expression profiling data sets (GSE10072, GSE116959, GSE31210, GSE32867, GSE7670, GSE32863, GSE75073 and GSE72094) were obtained from GEO database (https://www.ncbi.nlm.nih.gov/gds). Of the eight microarray data sets, GSE72094 has detailed clinical prognostic information, so it is used as a validation set to participate in the study, and the other seven sets of data sets are used to study the differential expression of genes. The CLEC10A protein expressed level in lung tissue was explored based on the immunohistochemistry data from HPA (Human Protein Atlas) database (https://www.proteinatlas.org/).

### 2.2 Differential expression analysis of CLEC10A

The Wilcox test and Kruskal test were applied to assess the differential expression of CLEC10A.

### 2.3 Linked omics database analysis

The LinkedOmics database (http://www.linkedomics.org/login.php) is utilized to analyse 32 TCGA cancer‐associated multidimensional data sets. The differentially expressed genes related to CLEC10A were screened from the TCGA LUAD cohort through the LinkFinder module in the database, and the correlation of results was tested by the Pearson correlation coefficient and presented respectively in volcano plot and heat maps. Function module analysis of Gene Ontology biological process (GO_BP), Kyoto Encyclopedia of Genes and Genomes (KEGG) pathways by the gene set enrichment analysis (GSEA) in the LinkInterpreter module.

### 2.4 TIMER and ESTIMATE database analysis

Tumor IMmune Estimation Resource (TIMER) database (https://cistrome.shinyapps.io/timer/) is a comprehensive resource for systematic analysis of immune infiltrates across diverse cancer types, which includes 32 cancer types. TIMER used a deconvolution method to infer the abundance of tumour‐infiltrating immune cells (TIICs) from gene expression profiles of the LUAD samples in the TCGA and GEO dataset.

Estimation of STromal and Immune cells in MAlignant Tumor tissues using Expression data (ESTIMATE) database (https://bioinformatics.mdanderson.org/public‐software/estimate/) is an algorithm using gene expression data to generate Immunescore, which represents the degree infiltration of immune cells in tumour tissue.

### 2.5 TISIDB database analysis

The TISIDB database (http://cis.hku.hk/TISIDB) is a web portal for tumour and immune system interaction, which integrates multiple heterogeneous data types. They pertain to 988 reported immune‐related anti‐tumour genes, high‐throughput screening techniques, molecular profiling and para‐cancerous multi‐omics data, as well as numerous resources for immunological data gathered from seven public databases. Here, TISIDB provides us associations for CLEC10A with lymphocytes, immunomodulators and chemokines.

## 3 RESULTS

### 3.1 Decreased CLEC10A expression in LUAD

**FIGURE 1:**
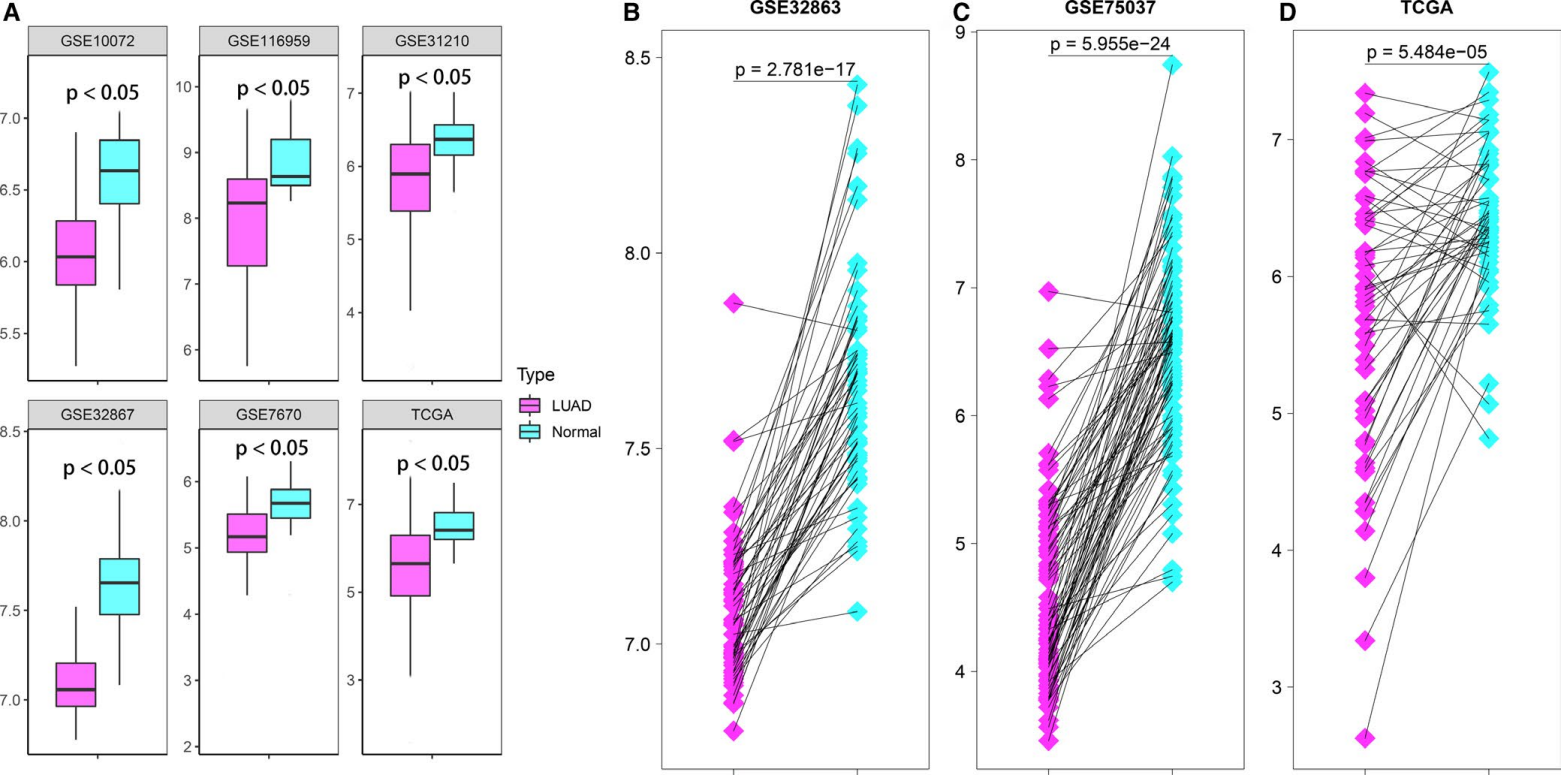
(A) Compared to normal lung tissue, CLEC10A was significantly down‐expression in LUAD tissue. (B‐D) CLEC10A was expressed at lower levels in LUAD compared to 192 pairs of non‐cancerous adjacent tissues from three different LUAD datasets

From six LUAD studies of GEO and TCGA database, CLEC10A expressed lowly in LUAD compared to non‐cancer tissues (Figure 1A). This comparison result was still accurate in 192 pairs of LUAD tissues and matched non‐cancer tissues in GEO and TCGA database (Figure 1B‐D). And the differential expression of CLEC10A between LUAD and normal tissues was also reflected in the protein expression level (Supplementary Figure 1). These results revealed that CLEC10A may play an inhibitory role in the LUAD process.

### 3.2 Correlations between CLEC10A expression and clinicopathological parameters in LUAD patients

**FIGURE 2:**
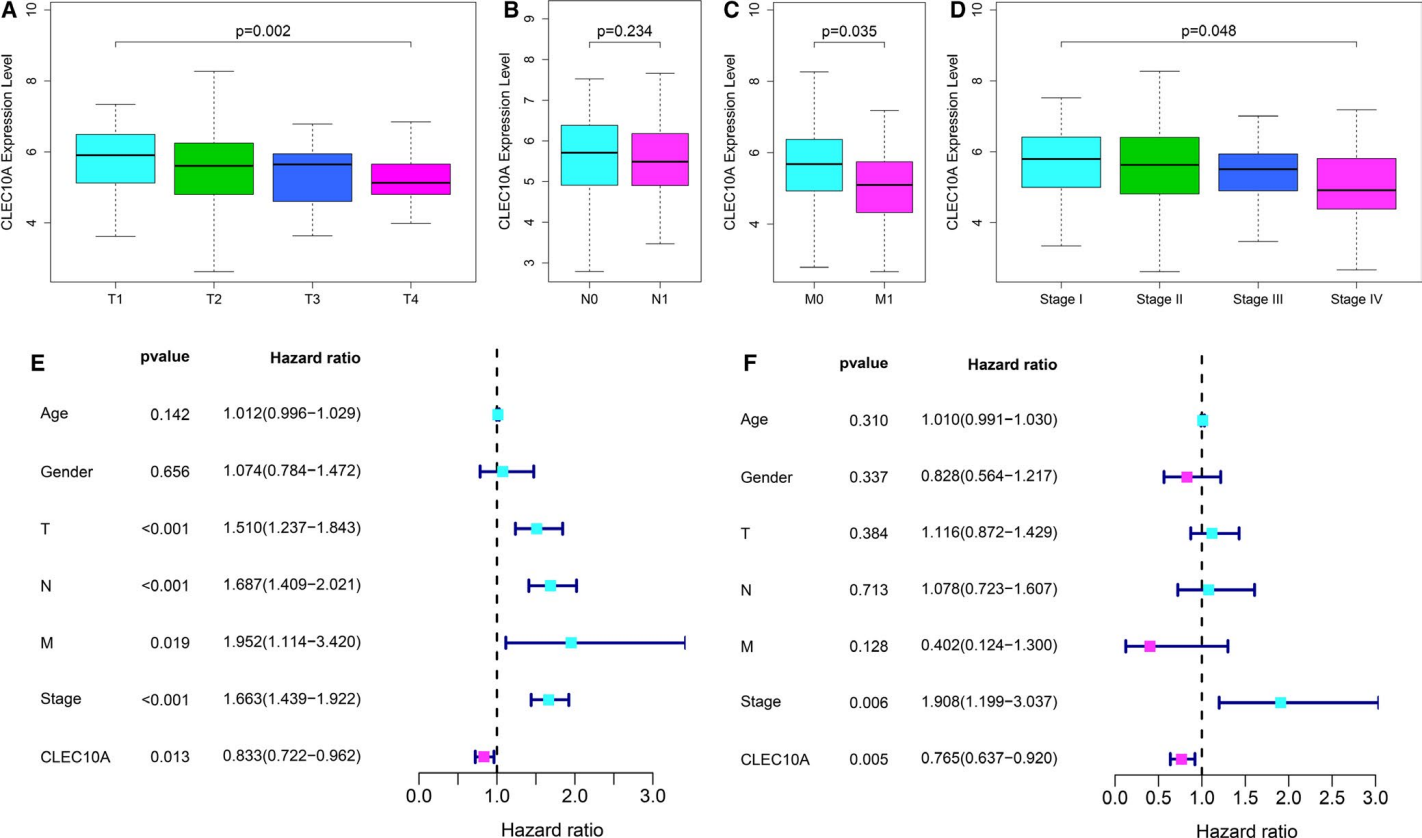
Association of CLEC10A expression with clinical parameters. (A) T stage; (B) N stage; (C) M stage; (D) TNM stage; (E‐F) Univariate and multivariate Cox analysis of CLEC10A and pathological parameters

Since the function of CLEC10A in LUAD remains unclear, the correlation research between the expression level of CLEC10A and clinicopathological features can help us reveal the role of CLEC10A in LUAD progress. The results showed that CLEC10A expression level significantly related to the stage alteration of T, M and TNM in TCGA LUAD data (Figure 2A,C,D). The higher CLEC10A expression could specifically lower T, M and TNM stage. Thus, CLEC10A was involved in delaying the progress of LUAD.

Furthermore, for understanding the prognostic role of CLEC10A expression in LUAD, the Cox proportional hazard regression model was employed to analyse prognostic factors. All LUAD patients were categorized according to the median CLEC10A expression value (high CLEC10A expression group and low CLEC10A expression group). The univariate analysis indicated that low CLEC10A expression was associated with the worse overall survival time. Other clinical parameters, such as advanced T stage, N stage, M stage and TNM stage, also correlated with the worse overall survival time (Figure 2E). For verifying the prognostic value of CLEC10A in LUAD, we performed the multivariate analysis. The results showed that only CLEC10A expression and TNM stage were independently associated with the overall survival time (Figure 2F), which shows that CLEC10A expression can not only participate in guiding clinical work like common clinical phenotypes, but also means that its role in evaluating patients' clinical prognosis is superior to T stage, N stage and M stage.

### 3.3 CLEC10A Co‐expression network in LUAD

**FIGURE 3:**
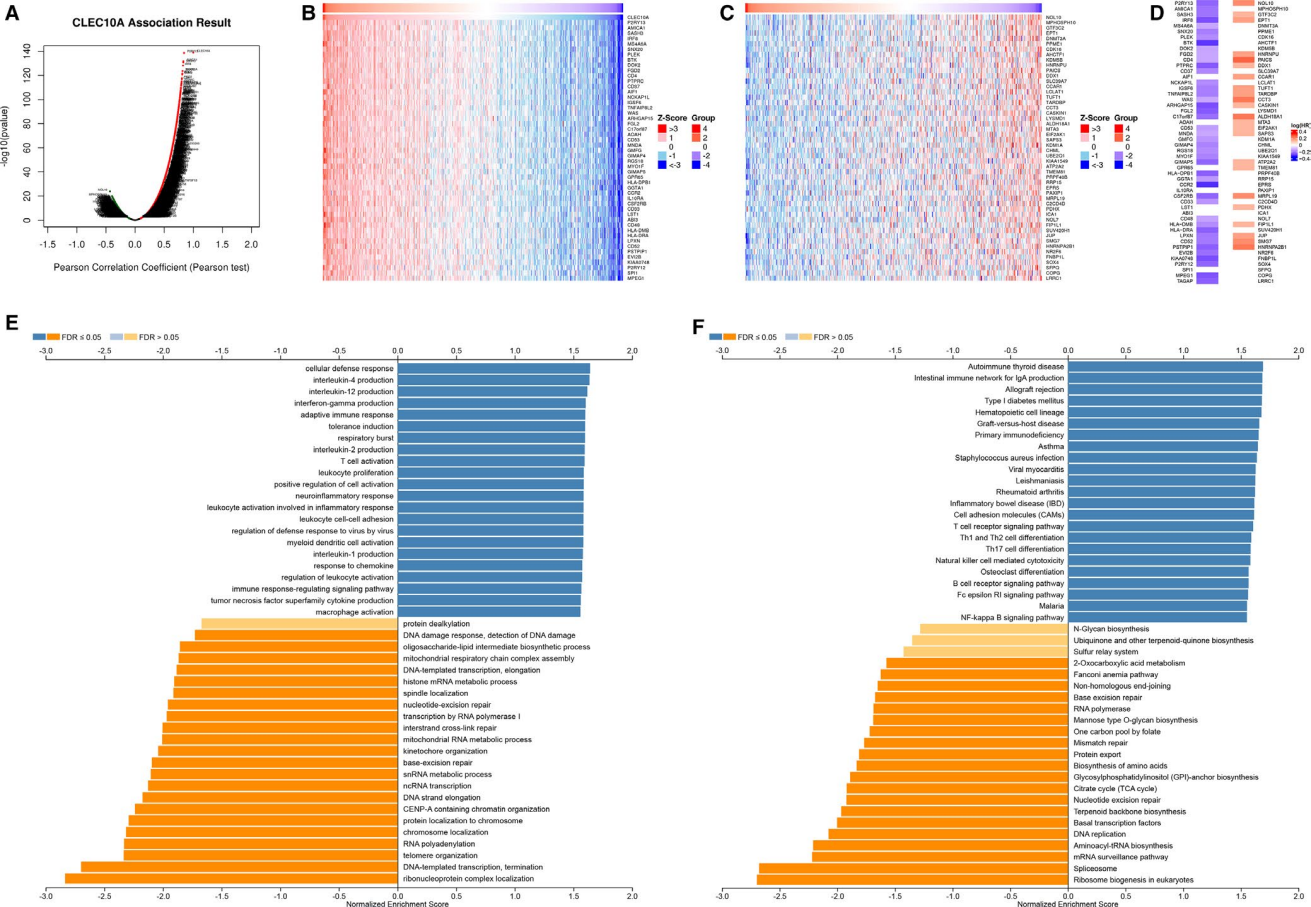
The co‐expression genes with CLEC10A in LUAD from the LinkedOmics database. (A) The whole significantly associated genes with CLEC10A distinguished by Pearson test in LUAD cohort. (B‐C) Top 50 genes positively and negatively related to CLEC10A in LUAD showed, respectively, by heat maps. Red represents positively linked genes and blue represents negatively linked genes. (D) Survival map of the top 50 genes positively and negatively associated with CLEC10A in LUAD. (E‐F) GO annotations and KEGG pathways of CLEC10A in LUAD cohort

For gaining the knowledge of CLEC10A biological function in LUAD, the LinkFinder module in the LinkedOmics web portal was deployed to check the co‐expression pattern of CLEC10A in TCGA‐LUAD. As is plotted in Figure 3A, it showed that 6116 genes (dark red dots) positively correlated with CLEC10A, and 4456 genes (dark green dots) negatively correlated. Figure 3B,C show the heat maps of the top 50 genes positively and negatively associatedwith CLEC10A, respectively. It is worth noting that the top 50 positively genes highly owned probability of becoming low‐risk markers in LUAD, of which 43/50 genes had protective hazard ratio (HR). In contrast, there were 23 of the top 50 genes with unfavourable HR in the top 50 negatively significant genes (Figure 3D).

GO term annotation showed that co‐expressed genes of CLEC10A join mainly in cellular defence response, interleukin‐4 production, interleukin‐12 production, interferon‐gamma production, adaptive immune response, tolerance induction, respiratory burst, interleukin‐2 production, T cell activation and leukocyte proliferation, etc (Figure 3E).

KEGG pathway analysis indicated enrichment in autoimmune thyroid disease, intestinal immune network for IgA production, allograft rejection, primary immunodeficiency, staphylococcus aureus infection, type I diabetes mellitus, graft‐versus‐host disease, asthma and hematopoietic cell lineage, etc (Figure 3F).

These results show that a wide influence of CLEC10A expression network on the prognosis and immune activation in LUAD.

### 3.4 Association between CLEC10A with immune infiltration level

**FIGURE 4:**
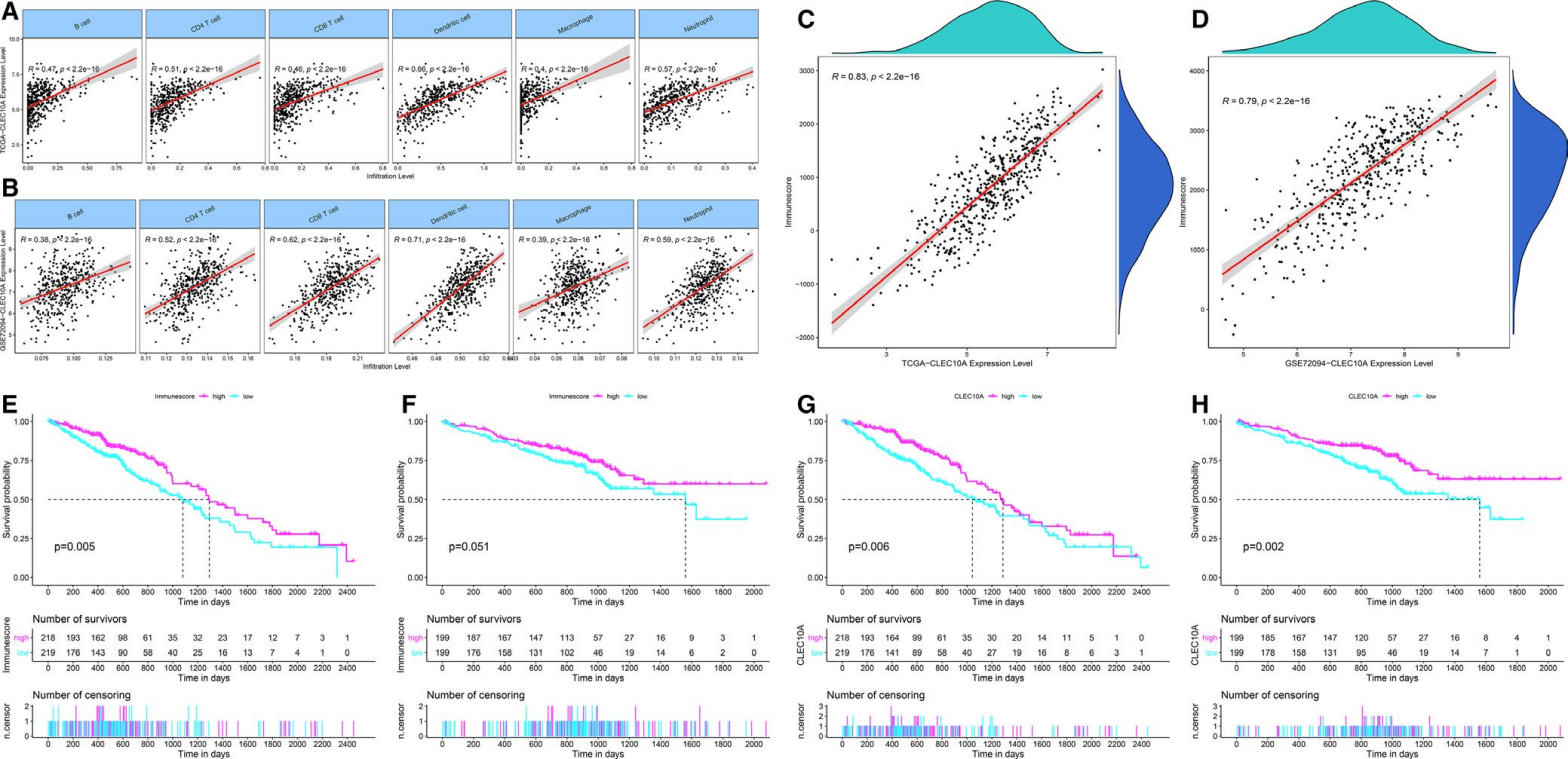
Associations between the CLEC10A expression and immune infiltration level in LUAD from the TIMER and ESTIMATE database. (A‐B) The expression levels of CLEC10A in the TCGA‐LUAD and GSE72094 dataset had a significant positive correlation with the infiltration level of B cells, CD4 T cells, CD8 T cells, dendritic cells, macrophages and neutrophils. (C‐D) The expression of CLEC10A has a significant positive correlation with the immune score of LUAD samples based on the ESTIMATE algorithm in the TCGA‐LUAD and GSE72094 dataset. (E‐F) Patients with higher immune scores had higher overall survival time in the TCGA‐LUAD and GSE72094 dataset. (G‐H) Patients with higher CLEC10A expression level had higher overall survival time in the TCGA‐LUAD and GSE72094 dataset

We explored whether CLEC10A expression can affect various immune cell infiltration levels in LUAD from the TIMER database. The significant positive associations between CLEC10A expression with B cells, CD4 T cells, CD8 T cells, dendritic cells, macrophages and neutrophils were all proved by Pearson correlation analysis (Figure 4A). The positive associations between CLEC10A expression with these immune cells in the TCGA‐LUAD dataset were also well confirmed in GSE72094 dataset (Figure 4B).

We then used the ESTIMATH algorithm to analyse whether CLEC10A expression linked to the infiltration level of total immunity in LUAD. The result shows that an obvious association between CLEC10A and Immunescore in both TCGA and GEO LUAD datasets (Figure 4C,D). Moreover, patients owned high Immunescore have a better overall survival time than patients with low one (Figure 4E,F), which is consistent with the prognostic results of single factor calculations that including only CLEC10A expression (Figure 4G,H).

### 3.5 Relation between CLEC10A with immune molecules

Finally, to broaden the cognition of the correlation between CLEC10A and immune infiltration, we investigated the connections between CLEC10A expression and various immune signatures, which included the immune‐related signatures of 28 TIL types from Charoentong's study, three kinds of immunomodulators, chemokines and receptors.

**FIGURE 5:**
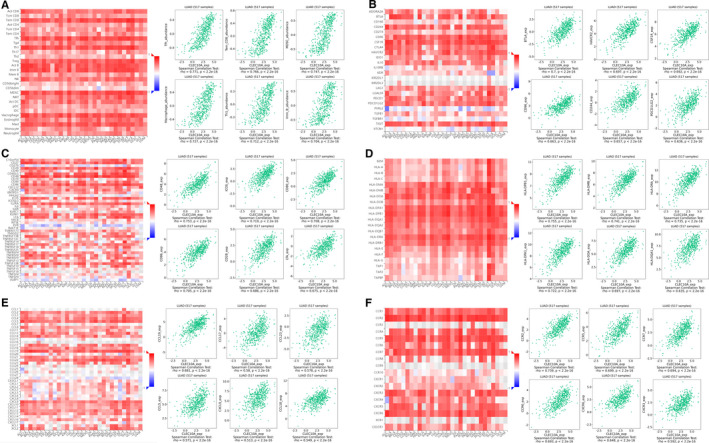
Associations of the CLEC10A expression level with lymphocytes, immunomodulators and chemokines in LUAD from TISIDB database. (A) Correlations between abundance of tumour‐infiltrating lymphocytes (TILs) and CLEC10A (plus the six TILs with the highest correlation). (B‐D) Correlations between immunomodulators and CLEC10A (plus the six immunomodulators with the highest correlation, respectively). (E‐F) Correlations between chemokines (or receptors) and CLEC10A (plus the six chemokines (or receptors) with the highest correlation, respectively)

Associations between CLEC10A expression and various immune signatures were obtained from the TISIDB database. Figure 5A shows the correlations between CLEC10A and tumour‐infiltrating lymphocytes (TILs), including Tfh_abundance, Tem_CD8_abundance, MDSC_abundance, Macrophage_abundance, Th1_abundance and Imm_B_abundance. Immunomodulators can be further separated into 3 groups containing immunoinhibitors, immunostimulators and major histocompatibility complex (MHC) molecules. Figure 5B shows correlations between immunoinhibitors including BTLA_exp, HAVCR2_exp, CSF1R_exp, CD96_exp, CD244_exp and PDCD1LG2_exp with CLEC10A. Figure 5C shows correlations between CLEC10A expression and immunostimulators, including CD48_exp, ICOS_exp, CD80_exp, CD86_exp, CD28_exp and LTA_exp. Figure 5D shows correlations between CLEC10A expression and MHC molecules, including HLA‐DPB1_exp, HLA‐DMB_exp, HLA‐DRA_exp, HLA‐DPA1_exp, HLA‐DOA_exp and HLA‐DQA1_exp. Figure 5E shows correlations between CLEC10A expression and chemokines, including CCL19_exp, CCL17_exp, CCL23_exp, CCL5_exp, CXCL9_exp and CCL18_exp. Figure 5F shows correlations between CLEC10A expression and receptors, including CCR2_exp, CCR5_exp, CCR7_exp, CCR6_exp, CXCR6_exp and CXCR3_exp.

Therefore, it was confirmed that CLEC10A participating widely in modulating various immune molecules in LUAD to affect immune infiltration in the tumour microenvironment.

## 4 DISCUSSION

Tumour cells exist in a complex tumour microenvironment (TME).^12^ The essence of TME is the cellular and non‐cellular components present in and around the tumour. Generally, TME is subdivided into extracellular matrix (ECM), stromal cells and immune cells.^13^ ECM is a complex network composed of multiple components such as collagen, integrin, laminin, fibronectin, glycosaminoglycans, matrix metalloproteinases (MMP) and secreted cysteine‐rich acidic proteins, which provide structural support as well as biochemical reagents and biomechanical signals for the growth of cancer cells.^14^ Stromal cells include fibroblasts, mesenchymal stromal cells, pericytes and occasionally fat cells, which secreted multiple growth factors, MMPs and the various ingredients contained in the above ECM to promote cancer cell growth and migration.^15^ And the immune environment constructed by multiple immune cells has been considered to be crucial of tumour progression and the overall effectiveness of cancer treatment, including chemotherapy and radiotherapy, especially immunotherapy.^6^, ^7^, ^13^

However, in most cases, the main role of TME is immunosuppression, which blocked anti‐tumour immunity and sustain tumour progression.^14^ Immunosuppressive effect of TME regulated by all immune cell types with immunomodulatory activities.^12^ Within TME, macrophages tend to become tumour‐associated macrophages (TAMs) to drive tumour progression, invasion and metastasis^16^; tumour‐infiltrating dendritic cells also incline to promote immunosuppression and tolerance, rather than drive anti‐tumour immunity^6^; neutrophils recruited into tumour, prevalently polarize towards the N2‐subtype with pro‐tumoral functions.^6^ As the main performers of anti‐tumour responses, T cell–mediated immune response reduced by most tumours with multiple strategies, including inhibiting T cell transport to tumour, interfering with antigen‐presenting cells and effector T cells.^7^, ^14^ The most typical is that tumour cells express PD‐L1 or PD‐L2 ligand matched to the PD‐1 protein of T cells, make it cannot find the tumour and send a signal to the immune system to attack the tumour, and directly cause T cells exhaustion. Clinically, by blocking this event, that is, the application of anti‐PD‐1 and anti‐PD‐L1 antibodies can not only facilitate the T cells’ proliferation, but also restore their cytotoxic responses against tumour cells.^14^, ^17^ Therefore, under the immense progress and bright prospects of tumour immunotherapy, all factors that participating in the modulation of immune cells in TME are worthy of our consideration and research.

In the past decade, the C‐type lectin receptors (CLRs) have acquired rising attention due to their functions in fine‐tuning of innate and adaptive immunity. CLRs are a huge family of receptors, containing more 1,000 members, with diverse functions, including cell adhesion, complement activation, tissue remodelling, platelet activation, endocytosis, phagocytosis and innate immune activation.^18^, ^19^ CLRs contain multiple C‐type lectin‐like domains, which can specifically recognize specific glycosylated antigens,^20^ the form of most foreign antigen display derived from tumour cells or viruses.^21^ In innate immunity, CLRs are mainly expressed on antigen‐presenting cells (macrophages, neutrophils and dendritic cells (DCs)) and play a critical role in identifying diverse pathogens, such as fungi, bacteria, viruses and parasites.^22^ Activation of the innate immune system was a crucial basis for constructing an adaptive immune response. The binding of CLRs to ligands results in various cellular responses, including respiratory burst, production of cytokines and chemokines, and consequently shaping the adaptive immune responses.^23^, ^24^ CLRs‐mediated innate immune responses can direct the progress of cellular immunity including Th1, Th17 and CD8 cytotoxic T lymphocytes cells immune responses through triggering the production of multiple cytokines.^25^, ^26^, ^27^, ^28^ Furthermore, it has been recently recognized that the CLRs activation involved in developing of regulatory T cells to modulate the function of CD4 T cells.^29^ The immunomodulatory effect of CLRs on cellular immunity has been treated as a promising solution for future cancer treatment. Several CLRs agonists or antagonists have proved to be potential anticancer drug candidates. The most representative one is β‐glucan, agonist of dectin‐1 (dendritic cell‐associated C‐type lectin‐1). In murine model, the application of β‐glucan showed a significant tumour growth inhibitory effect.^30^, ^31^ Moreover, the combined application with anti‐tumour monoclonal antibodies (mAbs) can significantly enhance the anti‐tumour efficacy of the latter.^32^ The anti‐tumour application of β‐glucans has been initially carried out clinically and achieved good results. Compared with the single application of conventional chemotherapy, the combination application with β‐glucan can highly extend patients’ survival time including ovarian cancer and advanced gastric cancer.^33^, ^34^ Some mechanisms have been proposed to explain the curative effect improvement of combination treatment. One of them is that β‐glucan can induce innate and adaptive immune responses through the dectin‐1 dependent pathway to increase the anti‐tumour therapeutic effect. In addition, through DC‐SIGN, DNGR‐1 and DEC‐205 with appropriate adjuvants to target the delivery of tumour antigens can prevent the development of tumours or mediated eradication in transplanted mouse models.^9^, ^35^, ^36^, ^37^ The current research has obviously demonstrated the huge potential of the CLRs family as a target for future tumour immunotherapy drugs.

As the member of the CLRs, CLEC10A, like other members, has been determined to involve in improving the immune response activity of immune cells. CLEC10A recognizes and acts on tumour‐associated Tn antigens and effectively presents the antigens to CD4 T cells.^9^ Moreover, the binding of CLEC10A to tumour‐associated antigens carrying α‐N‐acetylgalactosamine can obviously increase antigen‐specific CD8 T cell activation.^10^ Effective tumour eradication requires tumour‐specific CD8 and CD4 T cells. The role of CLEC10A in improving the anti‐tumour activity of immune cells has clearly received people's attention and proposed it as a target for cancer immunotherapy.^11^ However, there are some reports that the affinity of CLEC10A binding to Tn antigens on tumour cells was too low to complete efficient engagement.^38^, ^39^ And, CLEC10A can function as a negative regulator of effector T cells by interacting with CD45.^40^ These inconsistent conclusions illustrate accurately the complex mechanism of CLEC10A role in tumour tissue.

In this work, we tried to reveal CLEC10A's function in LUAD's TME construction. To acquire more detailed perceptions into the potential functions of CLEC10A in LUAD, we performed the bioinformatics analysis of public data.

Analysis of transcriptome from more than 1,200 clinical samples including four geographic regions indicated that CLEC10A mRNA levels lower obviously in LUAD than in non‐cancer lung tissue, whether viewed from the overall sample or from the matched sample of a single patient. And the expression level of CLEC10A has a significant effect on the clinical parameters including primary tumour status, lymph node metastasis status and tumour pathological stages. Moreover, multivariate analysis further shows that CLEC10A expression was an independent factor for LUAD patient's prognosis. Therefore, our results indicated that the down‐regulation of CLEC10A occurs in most LUAD samples and participates in the pathological progression of LUAD tissue. As a potential prognostic marker, CLEC10A deserves further clinical verification.

LinkedOmics database analysis further pointed out, not only CLEC10A has a significant impact on the prognosis of LUAD patients, most genes co‐expressed with CLEC10A in LUAD, whether positively or negatively related, also have an obvious linked to LUAD patients’ prognosis. Moreover, these co‐expressed genes are significantly focused on immune‐related pathways, which coincide with the known CLEC10A function. All signs indicate that CLEC10A can take part in regulating the immune microenvironment to improve the prognosis of LUAD.

The analysis results of the TIMER database show this inference more visually, the CLEC10A expression level in the TCGA‐LUAD and GSE72094 dataset had a highly positive connections with the infiltration level of B cells, CD4 T cells, CD8 T cells, dendritic cells, macrophages and neutrophils. Besides, the expression of CLEC10A has an obvious positive association with the immune score of LUAD samples based on the ESTIMATE algorithm, and patients who are grouped based on immune score or CLEC10A expression also have a constant prognostic relationship. These clearly show that the increasing expression of CLEC10A could ameliorate the immune microenvironment of tumours in LUAD patients by raising the infiltration level of immune cells, thereby improving patients’ prognosis. And as shown in TIMER database analysis also shows that the expression level of CLEC10A can also affect the immune cell infiltration level of multiple cancer tissues, including adrenocortical carcinoma, bladder urothelial carcinoma, breast invasive carcinoma, cervical squamous cell carcinoma and endocervical adenocarcinoma, colon adenocarcinoma, esophageal carcinoma, head and neck squamous cell carcinoma, kidney chromophobe, kidney renal papillary cell carcinoma, brain lower grade glioma, liver hepatocellular carcinoma, lung squamous cell carcinoma, ovarian serous cystadenocarcinoma, mesothelioma, pancreatic adenocarcinoma, rectum adenocarcinoma, sarcomav, skin cutaneous melanoma, stomach adenocarcinoma, testicular Germ Cell Tumors, thyroid carcinoma, uterine corpus endometrial carcinoma and uterine carcinosarcoma (Supplementary Figure 2). This result clearly shows that CLEC10A has a wide range of effects on the infiltration of immune cells in TME of most cancers, and it is worth further investigation in the field of tumour immunotherapy.

We concluded that there is a possible prognostic molecular marker for poor survival in LUAD, called CLEC10A expression. Decreased CLEC10A expression leads to worsening of clinical features (primary tumour scope, distant metastasis, pathological stage of tumour and prognosis). CLEC10A is the member of CLRs family, which have been confirmed to be involved in the regulation of the migration of various immune cells. In this research, we revealed that the raising expression level of CLEC10A was obviously linked to the increasing infiltration level of various TIICs in tumour tissues. The immune microenvironment composed of these TIICs profoundly affects the prognosis of LUAD. Therefore, in our clinical work, we can try to evaluate the degree of malignancy of the patient by measuring the expression of CLEC10A in the surgical specimens of LUAD patients, predict the prognosis of the patient, and even better assess the status of immune microenvironment, and develop immunotherapeutic drugs targeting CLEC10A. We recommend strongly that researchers in the field of tumour immunology conduct further research on CLEC10A in LUAD to gradually elaborate the biological function of CLEC10A in the immune microenvironment and prognosis of LUAD patients.

## CONFLICT OF INTEREST

The authors declare that there are no conflicts of interest.

## AUTHOR CONTRIBUTIONS

S. Zeng, X. Xu and H. Shen: Study design. M. He: Data collection and major analysis. S. Zeng, X. Xu and H. Shen: Study supervision. Y. Han and C. Cai: Data analysis and data interpretation. P. Liu: Statistical analysis. M. He and Y. Chen: Manuscript draft. All authors read and approved the final manuscript.

## Supporting information

Figure S1

Figure S2
