# Supplementary figures and images for: CLEC10A is a prognostic biomarker and correlated with clinical pathologic features and immune infiltrates in lung adenocarcinoma
Source: J Cell Mol Med. 2021 Mar 2;25(7):3391–9. doi: 10.1111/jcmm.16416 (PMC8034442; doi:10.1111/jcmm.16416)

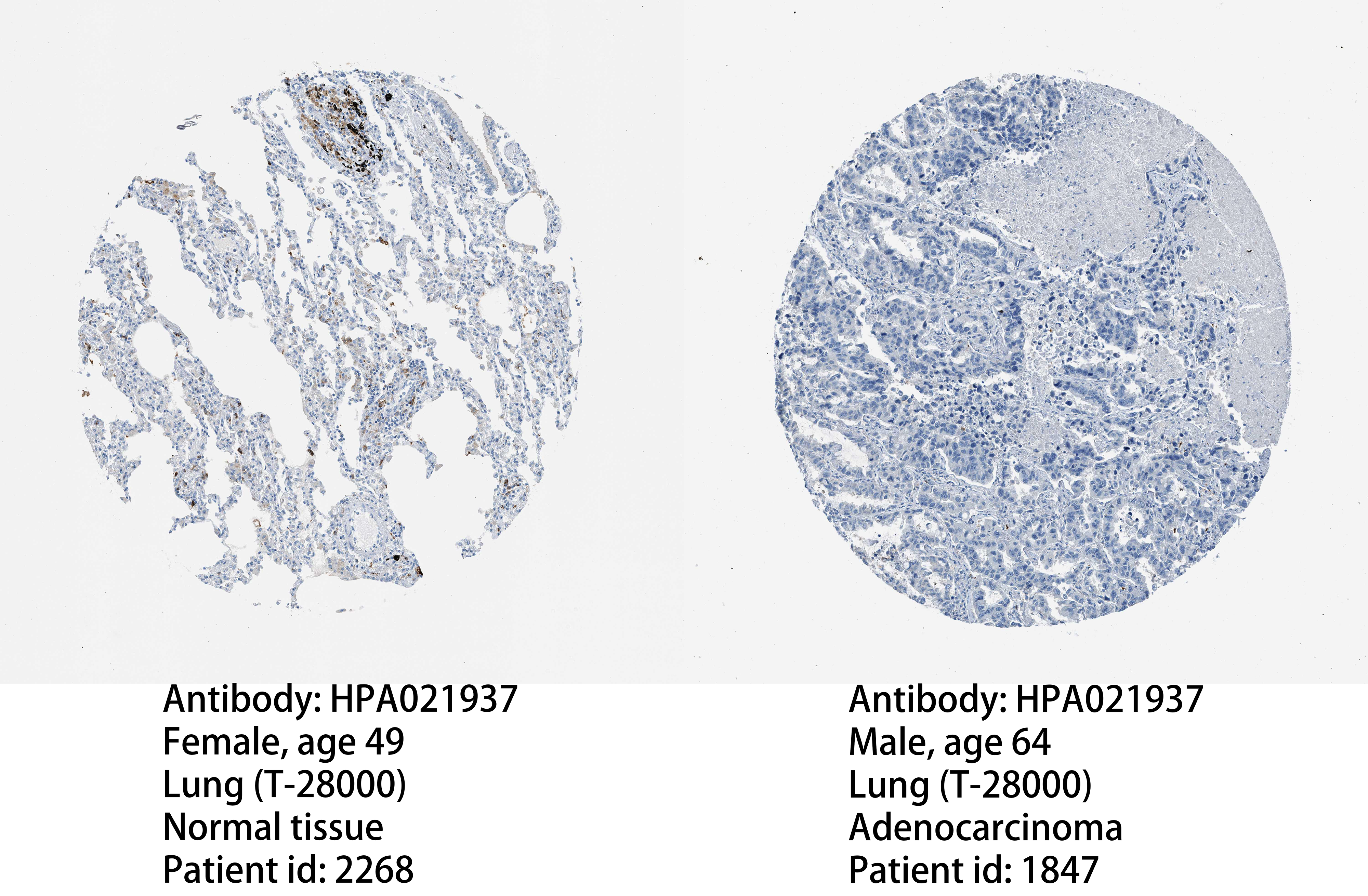

Supplement: Supplementary file 1 — Figure S1 [file JCMM-25-3391-s002.png]

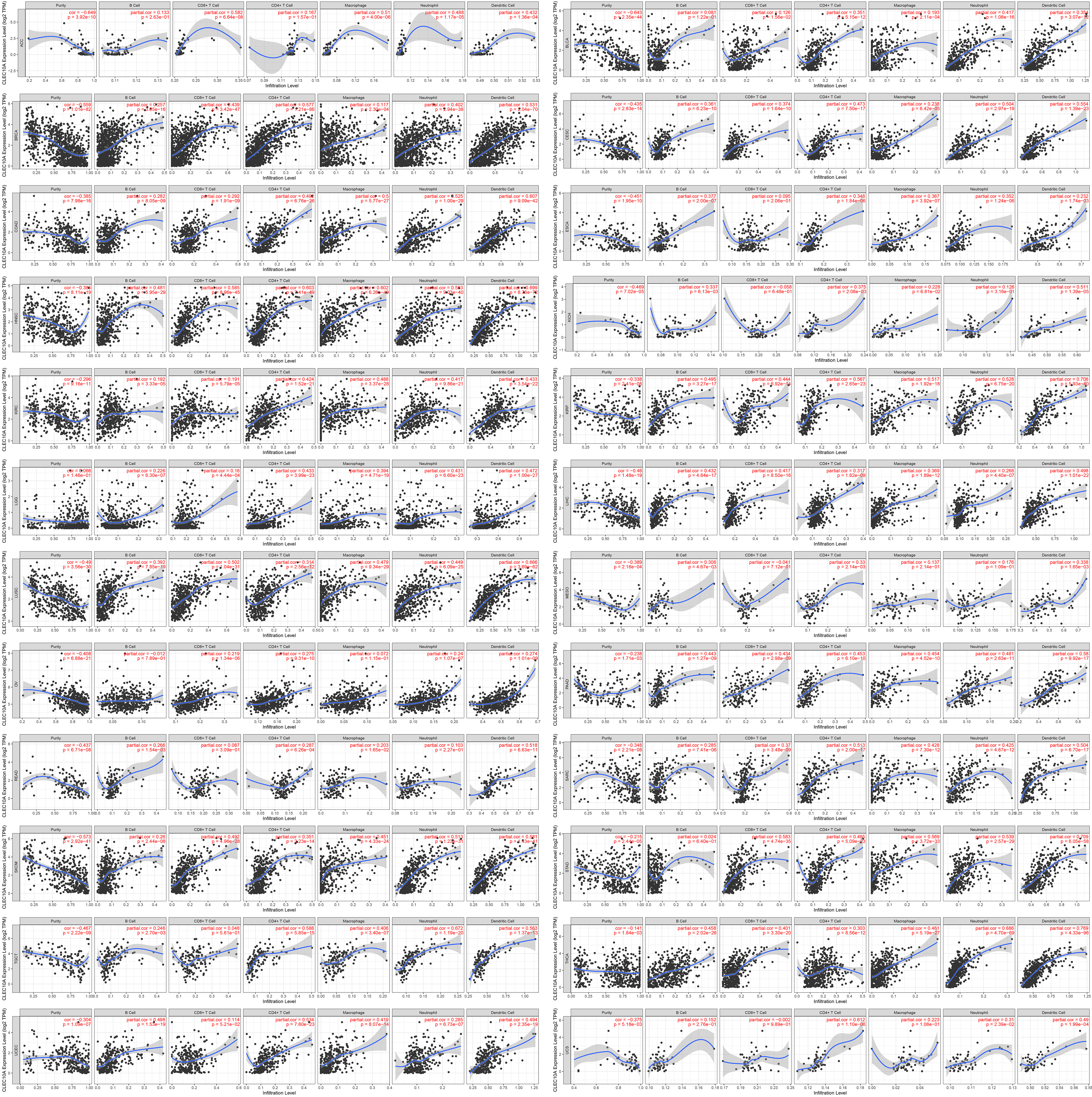

Supplement: Supplementary file 2 — Figure S2 [file JCMM-25-3391-s001.png]
